# Preconditioning process for dermal tissue decellularization using electroporation with sonication

**DOI:** 10.1093/rb/rbab071

**Published:** 2021-12-02

**Authors:** Min-Ah Koo, HaKyeong Jeong, Seung Hee Hong, Gyeung Mi Seon, Mi Hee Lee, Jong-Chul Park

**Affiliations:** 1 Cellbiocontrol Laboratory, Yonsei University College of Medicine, 50-1 Yonsei-ro, Seodaemun-gu, Seoul 03722, Republic of Korea; 2 Department of Medical Engineering, Graduate School of Medical Science, Brain Korea 21 Project, Yonsei University College of Medicine, 50-1 Yonsei-ro, Seodaemun-gu, Seoul 03722, Republic of Korea

**Keywords:** decellularization, electroporation, sonication, extracellular matrix

## Abstract

Decellularization to produce bioscaffolds composed of the extracellular matrix (ECM) uses enzymatic, chemical and physical methods to remove antigens and cellular components from tissues. Effective decellularization methods depend on the characteristics of tissues, and in particular, tissues with dense, complex structure and abundant lipid content are difficult to completely decellularize. Our study enables future research on the development of methods and treatments for fabricating bioscaffolds via decellularization of complex and rigid skin tissues, which are not commonly considered for decellularization to date as their structural and functional characteristics could not be preserved after severe decellularization. In this study, decellularization of human dermal tissue was done by a combination of both chemical (0.05% trypsin-EDTA, 2% SDS and 1% Triton X-100) and physical methods (electroporation and sonication). After decellularization, the content of DNA remaining in the tissue was quantitatively confirmed, and the structural change of the tissue and the retention and distribution of ECM components were evaluated through histological and histochemical analysis, respectively. Conditions of the chemical pretreatment that increase the efficiency of physical stimulation as well as decellularization, and conditions for electroporation and sonication without the use of detergents, unlike the methods performed in previous studies, were established to enable the complete decellularization of the skin tissue. The combinatorial decellularization treatment formed micropores in the lipid bilayers of the skin tissues while removing all cell and cellular residues without affecting the ECM properties. Therefore, this procedure can be widely used to fabricate bioscaffolds by decellularizing biological tissues with dense and complex structures.

## Introduction

The development of 3D bioscaffolds with good biocompatibility, non-toxicity and a structure that enables cell viability for clinical and experimental use has been actively progressing in tissue engineering [1, 2]. Bioscaffolds provide cells with physiological conditions and structural properties that are similar to those of the extracellular matrix (ECM). Although ECM components, such as collagen and proteoglycans are used to make bioscaffolds that mimic the ECM microstructure, single molecules cannot play the role of the entire ECM. Further, replication is difficult owing to the complexity of the composition and structure of ECM [3].

Decellularization is the process of removing cells and some antigenic components from tissues and organs of animals or humans [4–7]. After decellularization, active and some non-antigenic ingredients, such as collagen, fibronectin, glycoproteins and polysaccharides are retained to maintain the activity and the structural characteristics of natural tissues and organs [3, 8, 9]. In addition, decellularization is essential for self-transplantation and other tissue engineering processes, as the removal of antigens and cellular components reduces the risk of side effects such as inflammation and immune rejection at the transplant site [10, 11]. Decellularization techniques are classified as enzymatic, chemical (e.g. acid, alkali or detergent) and physical methods (e.g. freeze–thaw and mechanical agitation) [12–17]. However, these methods cannot be used for all applications, and each tissue or organ may require the use of different decellularization agents and protocols. The selection of the most effective agents for the decellularization of specific tissues and organ depends on many factors including the tissue cellularity, density, lipid content and thickness [18, 19].

The importance of ECM derived from the natural skin as a scaffold biomaterial is gaining recognition in the field of tissue engineering owing to its ability to preserve active molecules that serve as signals for tissue regeneration [20–22]. Recently, the dermal matrix has been proposed as a suitable candidate for the regeneration of skin wounds [23]. In particular, decellularized dermal matrix is being increasingly utilized in tissue engineering applications in recent years [24]. The decellularization of human skin overcomes the limitations related to allograft transplantation, making it a suitable technique to develop dermal substitute with high biocompatibility and low immunogenicity [25, 26]. Although many studies on the decellularization and preservation of skin tissue have already been published, these procedures have not been fully established yet [1]. Skin tissue tends to be more difficult to decellularize than other tissues owing to its dense, complex structure and abundant lipid content [11, 27]. Hence, skin tissue requires high chemical concentration and a long treatment time to achieve decellularization [28]. To date, most methods to decellularize skin tissue involved long-term chemical treatments. Although these methods produce acellular tissues, harsh processing of tissues often damages their fine ECM architecture, resulting in a partial loss of ECM components and a substantial reduction in the beneficial biological properties of the materials [15, 17, 29]. Conversely, gentle procedures that preserve the ECM structures increase the risk posed by the remaining cellular components, possibly eliciting serious immune reactions after transplantation [28, 30]. Thus, the ideal method of decellularization should ensure the complete removal of cells and cell remnants while preserving the composition of ECM and the 3D architecture of native tissues. Compared with a single method, a combination of methods may achieve an improved effect in overcoming the abovementioned shortcomings. In this study, acellular ECM was obtained from human dermal tissue through a combination of both chemical and physical methods including electroporation and sonication.

## Materials and methods

### Specimen preparation

The human skin tissues used for the experiments were kindly supplied by L&C Bio (Seongnam, Republic of Korea). The specimens were then stored temporarily at −20°C until further usage. Prior to decellularization, the skin tissues were thawed and cut into ∼10 mm × 5 mm × 10 mm sections.

### Electroporation and sonication system

For the process of electroporation and sonication, the samples were placed in a mesh pocket to maintain their shape and to control the areas of stimulation. Plate-shaped platinum electrodes were placed in parallel on both sides of the electroporation chamber, which was made of a backlit material; the distance between the electrodes was fixed at 20 mm. A 52 ml solution of 1 M sodium chloride (NaCl) was filled inside the chamber, and both ends of the electrodes were connected to a power supply (Kikusui Electronics Corporation, Japan) to facilitate a constant current output. The sonication was done in an ultrasonic device available commercially (UCP-10, Jeio Tech Co., Korea). Using distilled water (DW), the samples in the ultrasonic water bath were fixed to a depth between 7 and 9 cm from the transducer; the ultrasonic power supplied was 300 W. The sonication process was carried out at a frequency of 40 kHz for a total of 2 h. The experiment was performed at a constant temperature of 35 ± 1°C.

### Quantitative measurement of DNA content

Double-stranded DNA (dsDNA) was extracted from native and decellularized tissue samples. The tissue samples were chopped finely, and a lysis buffer with proteinase K was added to the samples. The samples were incubated at 56°C in a water bath overnight. The lysed solutions were spun down to filter out tissue debris, and the supernatant was collected. DNA concentration was determined by measuring the absorbance at 260 nm using a Nanodrop Spectrophotometer (ND-1000, Nanodrop Technologies Inc.).

### Scanning electron microscopy

The tissue structure was confirmed by scanning electron microscopy (SEM). Briefly, the specimens were fixed for 24 h in Karnovsky’s fixative (2% glutaraldehyde, 2% paraformaldehyde in 0.1 M phosphate buffer, pH 7.4) and then washed two times for 30 min in 0.1 M phosphate-buffered saline (PBS). Specimens were post-fixed with 1% OsO_4_ for 2 h and dehydrated in an ascending gradual series (50∼100%) of ethanol using a Critical Point Dryer (Leica EM CPD300). Finally, the specimens were coated with platinum by ion sputter (Leica EM ACE600) and observed with a field emission SEM (Merlin, Zeiss, Germany).

### Histological and histochemical analyses

The native and decellularized tissues were fixed in 10% neutral-buffered formalin, processed using conventional histochemical techniques, embedded in paraffin wax, and then sectioned to obtain a thickness of 4 μm. The sections were deparaffinized by submerging into three series of absolute xylene and then hydrated in a graded ethanol series (100%, 90% and 70% to DW). The samples were then placed on slides and stained for histological or histochemical evaluation. Sections were deparaffinized, rehydrated and stained with picrosirius red (PSR), as previously described [31]. In addition, the sections were stained with hematoxylin and eosin (H&E), periodic acid–Schiff (PAS) Morel-Maronger modified and Verhoeffe van Giseon (VVG) using their specific staining kits (all from Bio-Optica, Italy) following the manufacturer’s protocol. H&E staining was used to evaluate the effectiveness of decellularization procedures and the architecture of the decellularized dermal tissue; PSR was used to detect collagen; and PAS and VVG were used to detect glycoproteins and elastic fibers, respectively. The sections were observed under an optical microscope (BX40, Olympus, Japan).

### Statistical analyses

All values are expressed as mean ± standard deviation of the mean. Differences between mean values of normally distributed data were assessed by Student’s *t*-tests. *P *<* *0.05 was considered statistically significant.

## Results and discussion

Decellularization techniques that enable the production of tissue engineering scaffolds close to the natural state have pioneered a new field in regenerative medicine [32]. These techniques are used to fabricate tissue-derived scaffolds and mimic the essential physical and chemical cues efficiently for cell adhesion, proliferation, migration, differentiation and restoration of function [33]. In this study, we developed a decellularization method that combines chemical and physical methods of decellularization.

**Figure 1.**
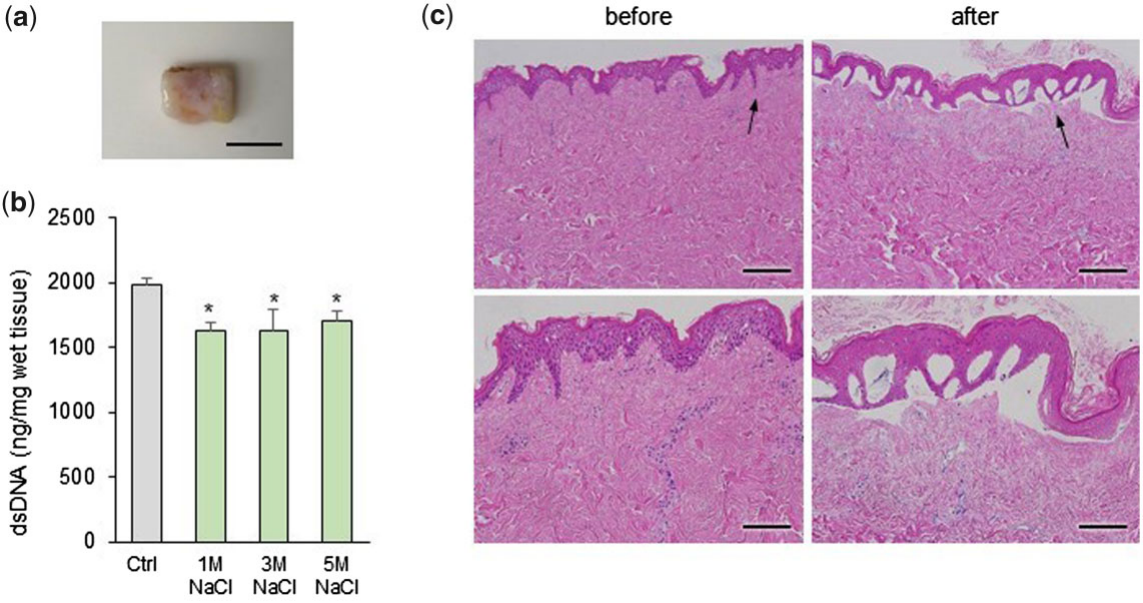
(**a**) Macroscopic image of human skin tissue specimens before decellularization. The samples were cut to size of ∼10 × 5 mm^2^, 10 mm thick sections. Scale bar: 1 cm. (**b**) Quantification of residual dsDNA in skin tissues after each immersion in 1, 3 and 5 M NaCl for 18 h. *P <* *0.05 vs. Ctrl. (c) Histological analysis of H&E-stained samples before and after 1 M NaCl treatment for 18 h (nuclei: dark violet, cytoplasm: pink or purple). Scale bar: 200 μm (upper), 100 μm (bottom). The magnified part is indicated by the black arrows

First, the effect of osmotic shock on the decellularization of skin tissue was confirmed. The skin tissue was taken from the back of the human body, the subcutaneous layer was trimmed, because of the dense structure of the tissue, to a thickness of 10 mm for facilitating decellularization (Fig.  1a). These specimens were washed by immersing in DW, a hypotonic solution. Afterwards, hypertonic NaCl at a concentration of 1–3 M was applied to weaken the cell membranes. The control group was treated with saline instead of NaCl. The residual DNA content within NaCl-treated samples was decreased in all groups compared to the control, but there was no significant difference according to the concentration (Fig.  1b). Therefore, 1 M NaCl was used in subsequent experiments. Figure  1c shows the changes observed by H&E staining of the skin tissues treated with hypertonic 1 M NaCl. As a result, the epidermis was detached from the dermis and was completely removed during the decellularization process. However, cell nuclei were observed in the dermal layer after hypo- and hypertonic shocks.

**Figure 2.**
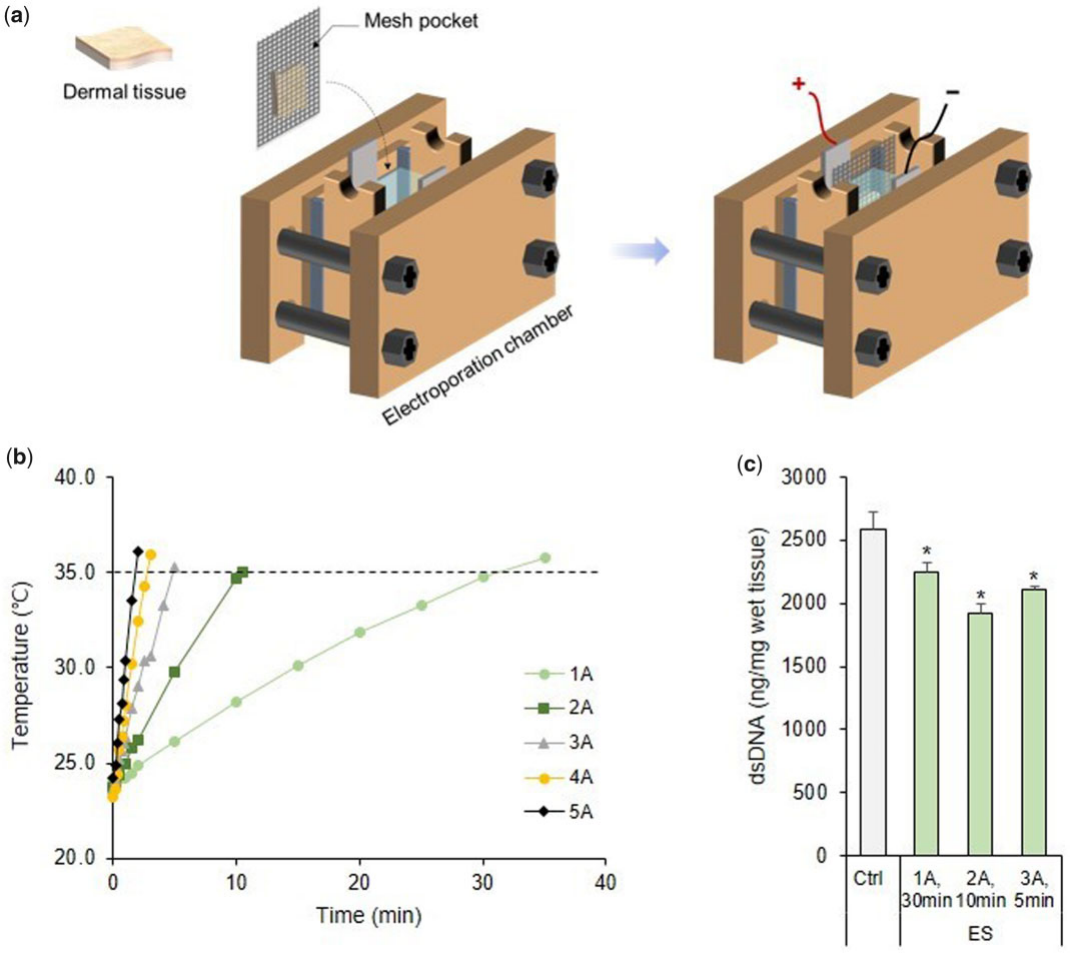
(**a**) Schematic diagram of the electroporation system used for tissue decellularization. (**b**) Measurement of temperature change of 1 M NaCl while applying ES. (**c**) Quantification of the residual DNA content in skin tissues after ES under conditions where the temperature of the 1 M NaCl solution did not exceed 35°C. *P <* *0.05 vs. Ctrl. dsDNA, double-stranded DNA

Tissue electroporation is a physical method that has been mainly used to decellularize small-sized soft tissues. Electroporation involves the application of an electric field across the cell that destabilizes the electric potential maintained by the cell membrane and results in the formation of nanoscale defects in the lipid bilayer [34]. Electrical stimulation (ES) is applied across a cell, destabilizing the electrical potential across the cell membrane, and resulting in that formation of irreversible nanoscale pores in the lipid bilayer and cell death due to loss of cell homeostasis [35, 36]. Studies have also used sonication in combination with sodium dodecyl sulfate (SDS) as a physical method for decellularization [37, 38]. SDS is mainly used in electroporation and sonication for decellularization, but this detergent is associated with certain drawbacks as it has a residual toxicity and might change the mechanical properties of tissues [1]. Herein, we used 1 M NaCl and DW for electroporation and sonication, respectively, to reduce the exposure time of the tissue to chemicals and achieve a high decellularization efficiency for obtaining a biological scaffold with preserved ECM. Additionally, we examined the conditions required for electroporation and sonication and the preparative conditions for treating the rigid skin tissue with chemical agents for complete decellularization. We fabricated a chamber for electroporation to enable the decellularization process (Fig.  2a). The temperature of the 1 M NaCl solution in the chamber was measured on the application of different current values (Fig.  2b). A condition where the temperature of the solution did not exceed 35°C was selected to prevent thermal damage of the ECM. The residual DNA in the wet tissue was quantitatively evaluated after applying the maximum current in the remaining conditions, except for those at 4 and 5 A, where the temperature change rapidly over time. As shown in Fig.  2c, the DNA content in the wet tissue did not decrease drastically compared with that in the control (Ctrl) tissue, but the content significantly decreased in all conditions after electroporation, especially when a 2 A current was applied for 10 min. Therefore, a 2-A current was applied for 10 min in subsequent experiments.

**Figure 3.**
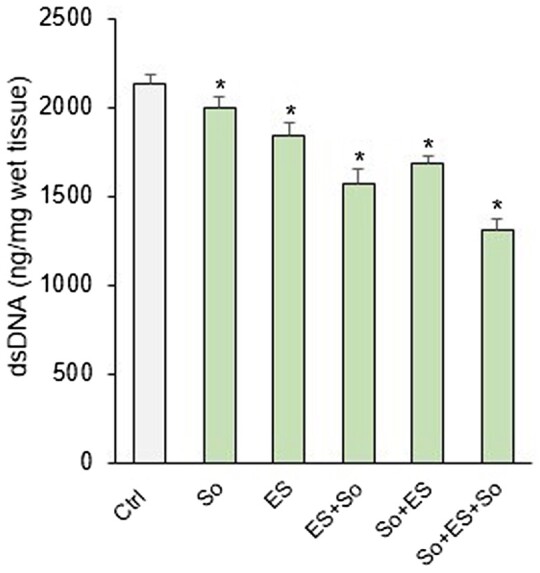
Comparison of changes in the amount of DNA remaining in the samples according to the processing sequence of ES and sonication (so) for 2 h. *P <* *0.05 vs. Ctrl. dsDNA, double-stranded DNA

Figure  3 is a comparison of the DNA content of the treated samples and the native skin (NS) under various electroporation and sonication conditions. The DNA content in the samples after the combination of electroporation and sonication was lower than that after electroporation or sonication alone. In addition, the DNA content decreased the most in the group for which sonication was performed for a total of 2 h, before and after ES. However, the use of physical stimulation, including ES and sonication alone, has limitations as it excessively reduces the DNA content of skin tissue. Alternatively, this result shows that a pretreatment process for enhancing the permeability of the target tissue must be performed in parallel when the tissue is decellularized using ES and sonication. Therefore, various chemical substances were used for pretreating the specimen before electroporation and sonication for effective decellularization.

**Figure 4.**
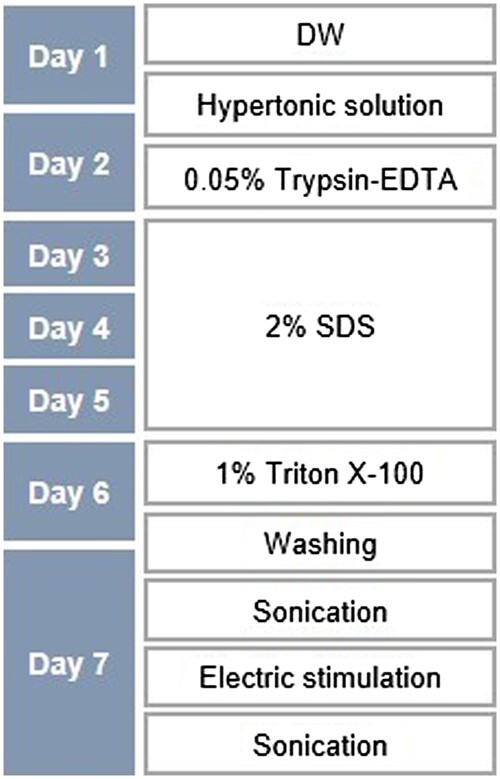
Flowchart depicting the process of tissue decellularization. The concentrations and treatment time of the pretreatment solutions for decellularization for electroporation and sonication are presented. DW, distilled water; SDS, sodium dodecyl sulfate

Figure  4 shows the detailed decellularization protocol where the preconditioning process involved sequentially treating samples with DW, 1 M NaCl, 0.05% (w/v) trypsin-EDTA and detergents, such as 2% (w/v) SDS and 1% (w/v) triton X-100 before electroporation and sonication. All samples were washed with PBS three times before carrying out further chemical treatments. Samples were treated with trypsin-EDTA at 37°C. All the chemical treatments, except for that involving trypsin-EDTA, were carried out at room temperature. Hypotonic and hypertonic solutions effectively lyse cells because of osmotic shock but do not remove cellular residues [13]. Trypsin is an enzymatic agent that cleaves peptide bonds on the C-side of Arg and Lys, effectively removing cellular components from the ECM. EDTA is a chelating agent that disrupts cells adhesion to the ECM by binding to metallic cations [13, 39]. Treatment using ionic detergents, such as SDS has the most potential for preparing biological scaffolds as these detergents lyse cytoplasmic and nuclear cellular membranes to aid decellularization [12]. Triton X-100 (non-ionic detergent) targets lipid–lipid and lipid–protein interactions but is ineffective in removing cellular materials. Nucleases (DNase and RNase) breakdown nucleic acids, but their products are difficult to remove from the ECM; these products may also invoke an immune response, impeding recellularization and inhibiting translational usage [40]. Nucleases were not used for decellularization because of these disadvantages.

**Figure 5.**
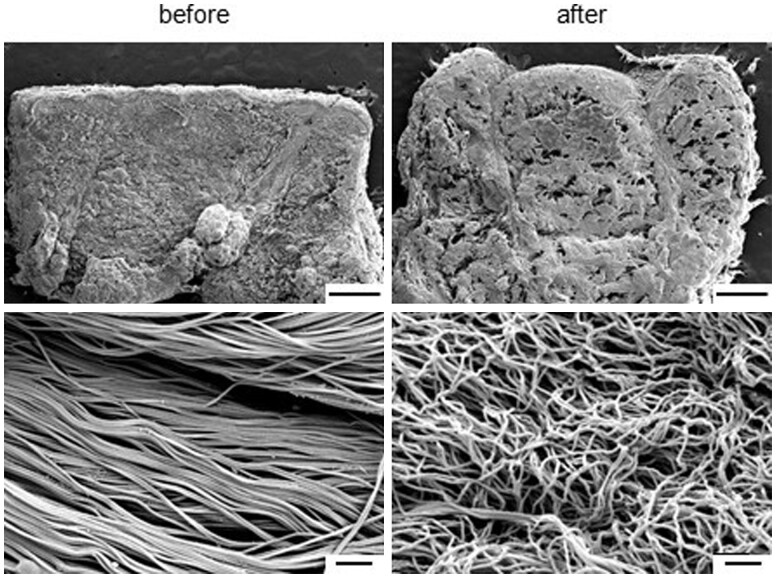
Representative SEM images of the cross-section of skin tissues before and after immersion in the pretreatment solution. The upper image shows the presence or absence of the epidermal layer, and the lower image shows the dermis. Scale bar: 1 mm (upper) and 1 μm (bottom). SEM, scanning electron microscopy

After conducting the pretreatment using various chemical agents in the order shown in Fig.  4, the structural changes of the tissue were confirmed through SEM observation. The low-magnification image in Fig.  5 shows the microcavities formed in the tissue; the high-magnification image shows the fiber bundles constituting the ECM of the treated tissue, which are loose when compared with those of the NS tissue. All decellularization protocol inevitably led to a certain degree of disruption in the matrix structure and orientation because they involve removal of all cellular components from the ECM [13, 33]. As a result, the formation of microcavities in the tissues during electroporation can help the penetration of ultrasonic waves and efficient transmission of ES into the tissue.

**Figure 6.**
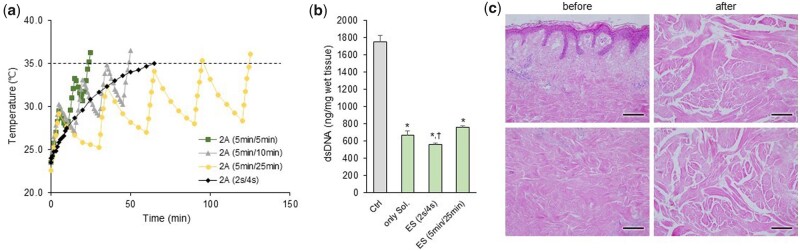
(**a**) Measurement of the temperature change of 1 M NaCl after pulsed ES, (**b**) quantification of residual DNA content in samples with sonication for 2 h+ES (2 A)+sonication for 2 h. *P <* *0.05 vs. Ctrl, ^†^*P *<* *0.05 vs. only chemical solution (Sol). (**c**) Representative images of H&E staining on native or decellularized skin. The top images show the upper part near the epidermis, and the lower images show the middle part of the dermis layer. Scale bar: 100 μm

Heat was generated during electroporation when 1 M NaCl was added to the inside of the chamber and the current was applied. The temperature was measured after applying the pulse currents to ensure that the electroporation conditions did not exceed 35°C (Fig.  6a and Table  1) to enhance the effect of ES. Pulsed current durations of 2 s ‘on’ and 4 s ‘off’ or 5 min ‘on’ and 25 min ‘off’ were utilized, after which the sonication was done for a total of 2 h (1 h before and after the ES of 2 A current). After the decellularization process was complete, the most obvious change in the tissues was that the original skin had lost its color and turned white (Supplementary Fig. S1). The content of residual DNA decreased the most under the condition of a pulse current time of 2 s/4 s, as shown in Fig.  6b. Additionally, H&E staining was done to evaluate the efficiency of our protocol in removing both cellular and nuclear materials from the tissue. Decellularization is not a quantitative indicator. However, tissues are considered decellularized if only a few cell nuclei are observed in tissue sections stained with 4',6-diamidino-2-phenylindole or H&E, based on studies that focused on structural remodeling reactions while avoiding harmful cellular and host reactions *in vivo* [13]. Besides, electroporation and sonication without the use of SDS can prevent the SDS used in the chemical pretreatment from remaining in the tissue. Figure  6c shows the SEM images that confirm the separation of the epidermis from the skin tissue and the formation of microcavities in the dermis. In addition, it was confirmed that the nuclei, skin appendages and adipose tissue seen in the NS were almost lost after decellularization (Supplementary Fig. S2).

**Table 1.** Under the pulsed ES application conditions, the total duration time required until just before the temperature of 1 M NaCl exceeds 35°C and the actual time that the ES is applied

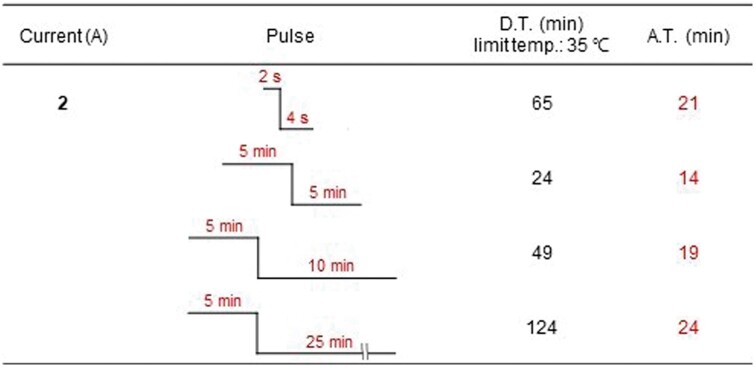

**Figure 7.**
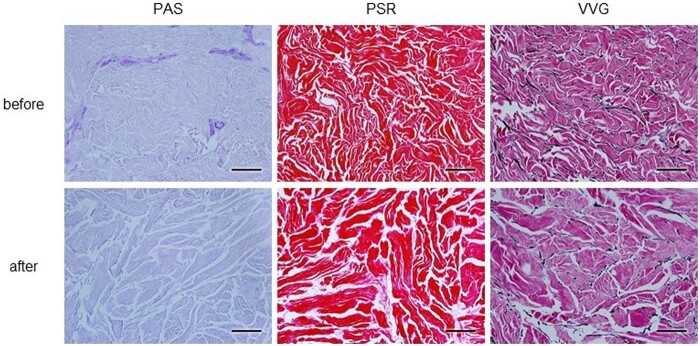
Representative images of PAS Morel-Maronger modified, PSR and VVG staining on cross-sections of native or decellularized skin. PAS stains the glycoproteins violet, PSR stains the collagen fibers red and VVG stains the elastic fibers black. Scale bar: 100 μm

PSR, PAS and VVG staining were used to evaluate the effect of decellularization on skin architecture and to assess the retention and distribution of ECM components in the decellularized tissue. Figure  7 shows the presence of collagen fibers, glycoproteins and elastic fibers that were not damaged in the decellularized samples, and it was confirmed that these components were similar to those in the NS, except there were wider gaps between the fibers in the decellularized samples. PAS staining showed that the counterstained cell nuclei were observed in the dermal layer before decellularization but not after decellularization.

We confirmed the removal of cells in the skin tissue through biochemical analysis and demonstrated that various ECM components are preserved in the decellularized tissue. As mentioned above, all decellularization processes result in unavoidable changes to the fibrous structure [41]. However, optimal decellularization methods should minimize the cellular and nuclear materials in the matrix without affecting consequently the mechanical properties of the tissue structure [41, 42]. In this respect, this article has a limitation in that there is no analysis result on the mechanical properties. Therefore, we will prove the cell affinity and mechanical properties of the decellularized tissue as further studies.

## Conclusion

This study proposed an optimized process to decellularize skin tissues completely by combining chemical and physical treatments, namely electroporation and sonication. We confirmed that the chemical treatment not only helps in the decellularization of the tissues but also loosens the collagen fiber bundles to produce a microscopically open porous matrix in the dermis and that the chemical treatment can be applied as a pretreatment that allows the transmission of physical stimuli to pass through the tissues. In addition, detergents were not used during this decellularization technique, which are generally used during electroporation and sonication in tissues, to eliminate their effects on the ECM architecture. Optimized conditions for pulsed current application and sonication were established to remove the cellular components from tissues. Consequently, the hybrid decellularization treatment formed micropores on the skin tissues while removing all cell and cellular residues and did not affect ECM properties. Therefore, this method can be widely used to fabricate bioscaffolds by decellularizing biological tissues having a dense and complex structure.

## Supplementary data

Supplementary data are available at *REGBIO* online.

## Funding 

This research was supported by the National Research Foundation of Korea (NRF) grant funded by the Korea government (MSIT) (No. 2017M3A9B3063638, No. 2019R1A2C2005256).

*Conflict of interest statement*. None declared.

## Supplementary Material

rbab071_Supplementary_Data
