# Supplementary material for: Preconditioning process for dermal tissue decellularization using electroporation with sonication
Source: Regen Biomater. 2021 Dec 2;9:rbab071. doi: 10.1093/rb/rbab071 (PMC9017362; doi:10.1093/rb/rbab071)
Supplement: rbab071_Supplementary_Data [file rbab071_supplementary_data.docx]

Supplementary data

Preconditioning process for dermal tissue decellularization using electroporation with sonication

Min-Ah Koo ^a,b^, HaKyeong Jeong ^a^, Seung Hee Hong ^a,b^, Gyeung Mi Seon ^a^, Mi Hee Lee ^a^, Jong-Chul Park ^a,b,^*

^a^ *Cellbiocontrol Laboratory,* ^b^ *Department of Medical Engineering, Graduate School of Medical Science, Brain Korea 21 Project, Yonsei University College of Medicine, Seoul 03722, Republic of Korea.*

* Corresponding author

Prof. Jong-Chul Park

Department of Medical Engineering,

Yonsei University College of Medicine,

50-1 Yonsei-ro, Seodaemun-gu,

Seoul 03722, Republic of Korea.

Tel: 82-2-2228-1917, Fax: 82-2-363-9923

E-mail: parkjc@yuhs.ac

**Supplementary Figures**


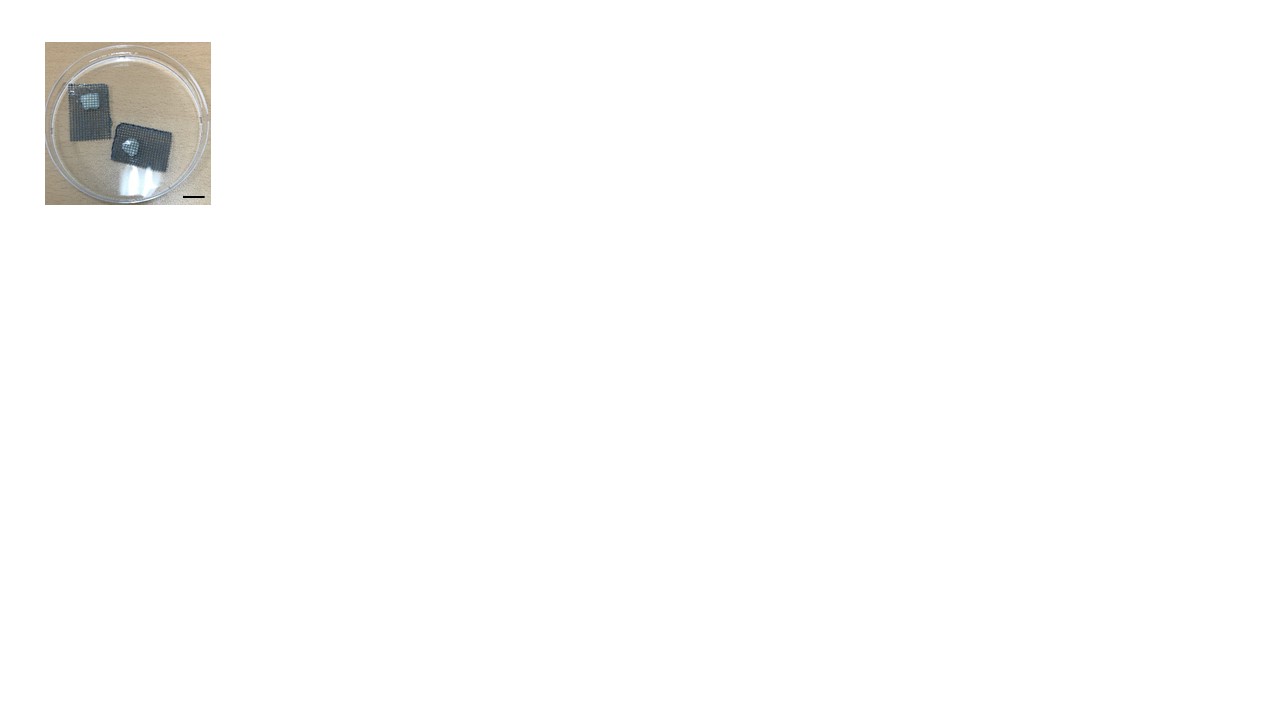


**Figure S1.** Macroscopic image of decellularized skin showing an obvious change in color after decellularization. Scale bar: 1.5 cm


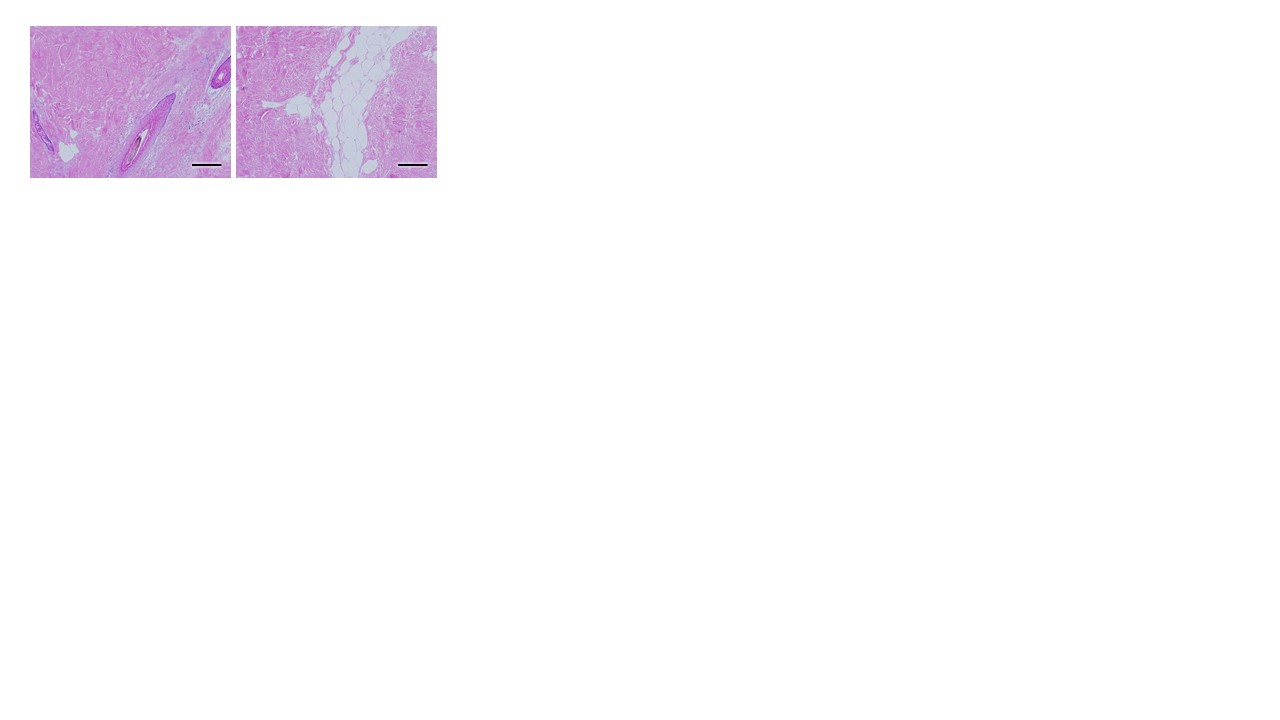


**Figure S2.** Images of hematoxylin and eosin-stained decellularized dermis show the presence of the cellular components and dermis appendixes before decellularization. Scale bar: 100 μm
